# The Epigenome of *Schistosoma mansoni* Provides Insight about How Cercariae Poise Transcription until Infection

**DOI:** 10.1371/journal.pntd.0003853

**Published:** 2015-08-25

**Authors:** David Roquis, Julie M. J. Lepesant, Marion A. L. Picard, Michael Freitag, Hugues Parrinello, Marco Groth, Rémi Emans, Céline Cosseau, Christoph Grunau

**Affiliations:** 1 Université de Perpignan Via Domitia, Perpignan, France; 2 CNRS, UMR 5244, Interactions Hôtes-Pathogènes-Environnements (IHPE), Perpignan, France; 3 Oregon State University, Corvallis, Oregon, United States of America; 4 CNRS, GenomiX IBiSA, Montpellier, France; 5 Leibniz Institute for Age Research—Fritz Lipmann Institute, Jena, Germany; Federal University of Minas Gerais, BRAZIL

## Abstract

**Background:**

Chromatin structure can control gene expression and can define specific transcription states. For example, bivalent methylation of histone H3K4 and H3K27 is linked to poised transcription in vertebrate embryonic stem cells (ESC). It allows them to rapidly engage specific developmental pathways. We reasoned that non-vertebrate metazoans that encounter a similar developmental constraint (*i*.*e*. to quickly start development into a new phenotype) might use a similar system. Schistosomes are parasitic platyhelminthes that are characterized by passage through two hosts: a mollusk as intermediate host and humans or rodents as definitive host. During its development, the parasite undergoes drastic changes, most notable immediately after infection of the definitive host, *i*.*e*. during the transition from the free-swimming cercariae into adult worms.

**Methodology/Principal Findings:**

We used Chromatin Immunoprecipitation followed by massive parallel sequencing (ChIP-Seq) to analyze genome-wide chromatin structure of *S*. *mansoni* on the level of histone modifications (H3K4me3, H3K27me3, H3K9me3, and H3K9ac) in cercariae, schistosomula and adults (available at http://genome.univ-perp.fr). We saw striking differences in chromatin structure between the developmental stages, but most importantly we found that cercariae possess a specific combination of marks at the transcription start sites (TSS) that has similarities to a structure found in ESC. We demonstrate that in cercariae no transcription occurs, and we provide evidences that cercariae do not possess large numbers of canonical stem cells.

**Conclusions/Significance:**

We describe here a broad view on the epigenome of a metazoan parasite. Most notably, we find bivalent histone H3 methylation in cercariae. Methylation of H3K27 is removed during transformation into schistosomula (and stays absent in adults) and transcription is activated. In addition, shifts of H3K9 methylation and acetylation occur towards upstream and downstream of the transcriptional start site (TSS). We conclude that specific H3 modifications are a phylogenetically older and probably more general mechanism, *i*.*e*. not restricted to stem cells, to poise transcription. Since adult couples must form to cause the disease symptoms, changes in histone modifications appear to be crucial for pathogenesis and represent therefore a therapeutic target.

## Author Summary

The blood fluke *Schistosoma mansoni* causes intestinal bilharzia. The parasite has a complex life cycle in which a freshwater snail serves as intermediate host from which the human infecting larvae hatch. These larvae will actively seek skin contact, penetrate through the epithelium and start developing straight away into adult worms. Development from larvae into adults needs thorough adjustment of gene expression through repositioning or modification of proteins that are associated with DNA (the chromatin). We decided to compare the chromatin of human infective larvae (cercariae), the first developmental stage after infection of the vertebrate host (schistosomula) and adults of *S*. *mansoni*. We found that cercariae possess chromatin structures (modifications of histone H3) around the beginning of genes that are very different from schistosomula and adults. We conclude that this structure serves to keep gene transcription in a poised state, *i*.*e*. transcription is initiated and can start immediately when the blocking histone modification is removed. A similar type of histone modification was found in embryonic stem cells of vertebrates and our data indicate that it is either a more ancient and/or more general means to poise transcription than previously assumed. Since many parasites possess infective stages that develop rapidly within the host, this particular chromatin structure could be a therapeutic target for a new class of antiparasitic drugs.

## Introduction

Histones are closely associated with DNA. Amino acids in their side-chains carry several modifications, such as methylation, acetylation and others. Specific combinations of histone modifications are associated with transcriptionally permissive or repressive chromatin structures, thus controlling gene expression at the transcriptional level [1]. Acetylation of histone H3 at lysine 9 (H3K9ac) is a mark associated with euchromatin and facilitation of transcription [2–6]. In model species the main enrichment of this mark is observed between the beginning of promoters and the 5’ end of genes and sometimes in enhancers [2,3,7]. In contrast, methylation at the same position (H3K9me3) is a landmark of constitutive heterochromatin. It is often highly enriched at telomeric, pericentromeric and repeat-rich regions, and believed to be responsible for stable large-scale repression [8–11]. In *Plasmodium falciparum* for instance, it is found in subtelomeric and some small intrachromosomal regions containing almost exclusively clusters of genes involved in antigenic variations [6]. In human, it was shown that this modification is mutually exclusive with trimethylation of histone H3 at lysine 4 (H3K4me3) on a given nucleosome [12]. H3K4me3 is often associated with transcription start sites (TSS), while trimethylation of H3 at lysine 27 is generally found in transcriptionally repressive heterochromatin. Consequently, the discovery of methylation at both sites (bivalent methylation) in several hundred genes of embryonic stem cells has attracted wide attention [13]. Embryonic stem cells (ESCs) are generated from the inner cell mass of mammalian blastocysts. These cells are self-renewing and can give rise to all lineages of the developing organism. The current view of bivalent methylation is that histone H3 trimethylated at lysine 27 (H3K27me3) represses transcription of lineage control genes during pluripotency, while H3K4me3 maintains them poised, *i*.*e*. transcription machinery is in position at the TSS, ready for activation upon reception of a signal that triggers differentiation (reviewed in [14]). When ESCs initiate differentiation, genes marked with bivalent trimethylation have two possible outcomes: H3K27me3 is removed and transcription occurs, or H3K4me3 is removed and genes stay silent. Since histones and histone modifications are extremely conserved through all kingdoms, we wondered if H3K9 methylation/acetylation and bivalent H3K4/K27 trimethylation might be a general mechanism by which a transient developmental state is maintained until development and differentiation into the next developmental state is triggered. Parasites are one of the models of choice to study this question since many of them have life cycles with very strong phenotypic differences between the stages, they are phylogenetically only distantly related to vertebrates and the environmental triggers are well-known, even though the molecular bases of switching to a new developmental stage are mostly unknown. Here we used the platyhelminth trematode *Schistosoma mansoni*. *S*. *mansoni* is the causative agent of intestinal bilharzia, a disease affecting 67 million people [15]. In contrast to many other parasites, the stage of the parasite infecting humans is actively seeking skin contact and penetrates the epidermis by use of cytolytic enzymes secreted from the pre- and post-acetabular glands and mechanical force. During skin penetration, cercariae lose their tails and undergo a drastic morphological and physiological transformation. Within two hours, the free-living larvae become obligatory endoparasitic schistosomula [16] that develop into adult worms, which reproduce in the blood stream of the host. Cercariae have therefore a double function: they are a vehicle for the parasites heritable information, but also true free-living animals with highly organized structures such as a nervous system and sensory organs, a digestive tract, excretory bladder and ducts, and a gland cell network. In addition to this, they have the capacity to develop very rapidly into full-grown adult worms, which are phenotypically very distinct from the cercariae.

The core histone isoforms of *S*. *mansoni* are canonical with 100% identity to human H4.A, 79–99% identity to H3 forms and 62–92% to H2A and H2B [17]. The distribution of histone modifications in *S*. *mansoni* was completely unexplored, and we decided to study the parasite epigenome by chromatin immunoprecipitation followed by sequencing (ChIP-Seq) of DNA associated with the above-mentioned histone isoforms H3K4me3, H3K9ac, H3K9me3 and H3K27me3. The genome of *S*. *mansoni* has been recently assembled and most of the gene structure data comes from automatic annotation [18]. Since gene prediction algorithms often miss the first exon (especially if they are untranslated), we performed RNA-Seq to unambiguously identify transcriptional start sites (TSS) and the expression status of genes.

## Methods

### Ethics statement

Housing, feeding and animal care followed the national ethical standards established in the writ of February 1st, 2013 (NOR: AGRG1238753A) setting the conditions for approval, planning and operation of establishments, breeders and suppliers of animals used for scientific purposes and controls. The French Ministère de l’Agriculture et de la Pêche and French Ministère de l’Éducation Nationale de la Recherche et de la Technologie provided permit A 66040 to our laboratory for experiments on animals and certificate for animal experimentation (authorization 007083, decree 87–848) for the experimenters.

### Animal breeding and preparation of parasites

The *S*. *mansoni* strain *Sm*Bre, originally sampled in Brazil was used in this study. The strain was maintained in its sympatric intermediate host strain *Bg*BRE of the mollusk *Biomphalaria glabrata*, and *Mus musculus* as definitive vertebrate host. Cercariae were collected from infected snails 30 days after infection by pipetting from spring water and sedimentation on ice. For isolation of adults, infected mice were killed after 60 days of infection by a lethal intraperitoneal injection of sodium pentobarbital, and adult worms were recovered by retrograde perfusions of the hepatic portal system with citrate (7.5%) saline (8.5%) solution administrated through the left ventricle [19]. Worms trapped in the liver or mesenteric system were collected after excising these organs.

*In-vitro* transformation of cercariae to schistosomula was done as described before [20]. The shells of infected snails were cleaned to remove rotifers, transferred to de-chlorinated water (about 75 mL for 10 snails), and exposed to a light source (60 W) for two hours in order to promote cercariae emission. Cercariae were gently collected with a 1 mL micro-pipet previously moistened, avoiding snail feces and mucus. They were transferred to a sterile 50 mL conical tube and placed on ice in the dark. After 30 minutes, cercariae were concentrated by centrifugation (400×g/10 minutes/4°C) and the supernatant was removed, leaving 5 mL of liquid. Five milliliters of phosphate buffered saline, supplemented with 200 U/mL of penicillin and 200 μg/mL of streptomycin (PBS-PS) were added and put at 37°C. The tails of cercariae were sheared by vortexing at maximum speed during one minute and subsequently centrifuged (400×g/1 minute/4°C). All further handling was done under sterile conditions. The supernatant, containing only tails, was completely removed and discarded. The pellet was resuspended in 2 mL PBS-PS. Schistosomula were separated from tails by centrifugation along a gradient of polyvinylpyrolidone-coated colloidal silica particles (Percoll) [21] prepared in 15 mL conical tubes (6 mL of the Percoll [Sigma, P4937], 1 mL 10 fold PBS, 0.25 mL 100 mM HEPES, 0.25 mL penicillin/streptomycin, 2.5 mL sterile water). One milliliter of the schistosomula suspension was applied to each column without disturbing the surface of the Percoll gradient. The preparation was centrifuged at 450×g for 10 minutes, with weakest possible brake. The top layer containing the tails was removed, and the pellet containing schistosomula was resuspended and washed twice in 10 mL of Dulbecco's modified Eagle's medium (DMEM) with 200 U/mL of penicillin, 200 μg/mL of streptomycin and 10 mM HEPES at 37°C (centrifugation 400×g/1 minute/4°C). Finally, schistosomula were resuspended in modified Basch's medium without antimycotics [20,22] and maintained at 37°C under 5% CO_2_ for 21 hours.

### RNA extraction and reverse transcription, quantitative PCR, and native chromatin immunoprecipitation

As the genome annotation of *S*. *mansoni* has mostly been done through prediction software, we performed RNA-Seq on cercariae to unambiguously identify TSS and exon-intron structures. Since published RNA extraction protocols for cercariae did not yield RNA suitable for Illumina sequencing, we developed the following method: 5,000 cercariae were ground with glass beads in liquid nitrogen for 5 minutes, resuspended in 0.5 mL TRIzol (Invitrogen), and 0.1 mL of chloroform was added. The tube was shaken vigorously for 1 minute and left at room temperature for 3 minutes, and was then centrifuged at ≥12,000×g for 15 minutes at 4°C. The upper phase was transferred to a new RNase-free tube, an equal volume of 70% ethanol was added and the tube was vortexed. PureLink RNA Mini kit (Ambion, 12183020) was used for further purification following the manufacturer’s protocol. RNA was recovered in 30 μL RNAsecure (Ambion, AM7006), left for 1 minute at room temperature, collected by centrifugation for 1 minutes at 8,000xg at room temperature and incubated at 65°C for 10 minutes. To remove remaining DNA, 3 μL of 10X TURBO DNase Buffer (TURBO DNA-free, Ambion, M1907) were added to 30 μL of total RNA in RNAsecure, followed by 3 μL of TURBO DNase (2 Units/μL). The preparation was gently mixed and incubated at 37°C for 30 minutes. DNase was removed with an RNA Clean-up system (RNeasy mini kit, QIAGEN, 74104) and eluted in 30 μL RNase-free water. For schistosomula, total RNA extraction, DNase treatment and purification methods were adapted from the protocol described above. 500 ng of this purified total RNA were reverse transcribed using identical concentrations (250 nmol/L) of random hexamers and oligo(dT)_18_ primers of Maxima H Minus Reverse Transcriptase kit (ThermoSCIENTIFIC). Quantitative PCR was performed using a LightCycler 480 System (Roche Diagnostics). We used 2.5 μL of cDNA (diluted 1/10 in water) in a total volume of 10 μL containing 1X LightCycler 480 SYBR Green I Master Mix (Roche Diagnostics) and 500 nmol/L of each primer. To ensure that a single product was generated, we separated amplification products on a Labchip GX DNA assay system (PerkinElmer). For each studied stage, the level of mRNA was normalized using the mean geometric transcription rate of three reference sequences (Smp_093230, Smp_197220 and Smp_089880) previously described by Wang and colleagues [23]. mRNA of Smp_034000 was measured using primers Smp034000.2F: GCGTGCTGTGGAGGAAAGCGA and Smp034000.2R: ACGCAGACAGGGCATCGTCA. The normalized relative quantities (NRQs) were calculated as described by Hellemans and colleagues [24], using equation 1 where E_target_ is the amplification efficiency of the gene of interest; E_ref_ is the amplification efficiency of the reference gene; ΔC_p,ref_ = C_p,ref_(calibrator)–C_p,ref_(sample); and ΔC_p,target_ = C_p,target_(calibrator)–C_p,target_(sample). For each reaction, the crossing point (Cp) was determined using the second derivative maximum method applied using Light Cycler Software version 3.3 (Roche Diagnostics). Male cercariae were arbitrarily chosen as calibrator. We obtained male cercariae by infecting host mollusk *B*. *glabrata* strain BgBre with single miracidium from SmBre strain. Sex determination was performed as described in [25]. Experiments were done in six biological replicates.

Native chromatin immunoprecipitation was done according to an earlier published protocol [26] and at least 1,500 cercariae, 2000 schistosomula or 1 adult couple using the following antibodies: H3K4me3 (Millipore, cat# 04–745 lot# NG1680351, 4 μL per reaction), H3K9Ac (Millipore, cat# 07–352 lot# DAM16924924, 8 μL per reaction), H3K9me3 (Abcam, cat# ab8898 lot# 733951, 4 μL per reaction), and H3K27me3 (Diagenode, cat# pAb-069-050 lot# A29900242, and cat# C15410069 lot# A1821D, 8 μL per reaction). For each sample, we used a control without antibody, to assess unspecific background (bound fraction) and input (unbound fraction). Inputs were used for normalization in all subsequent bioinformatics analyses. Experiments were done in duplicate. Antibodies were carefully tested for specificity as described in [27] and saturating quantities (see [27]) were used. Further details are available at http://methdb.univ-perp.fr/epievo/

### Illumina library generation and sequencing

For ChIPseq library construction, the ChIP sample preparation kit (Illumina Inc., USA) was used according to the manufacturer's protocol except for the indexed adapters. Those adapters came from the Truseq DNA sample preparation kit (Illumina Inc., USA). Briefly, 30 ng of DNA per condition was blunt ended and adenylated on its 3' ends. Illumina's indexed adapters were ligated to both ends of the adenylated DNA. Ligated DNA was size separated by electrophoresis through a 2% agarose gel and a size selection was performed at 400 bp. Once extracted from the agarose, size selected DNA was enriched by 18 cycles of PCR for 10 seconds at 98°C, 30 seconds at 60°C and 30 seconds at 72°C using Illumina's proprietary primers and Phusion DNA polymerase (New England Biolabs Inc., USA). Each library was verified using a DNA 1000 Chip on a Bioanalyzer 2100, quantified by real time PCR with the KAPA Library Quantification Kit for Illumina Sequencing Platforms (Kapa Biosystems Ltd, SA) and then diluted to 10 nM.

RNAseq library construction was done with the TruSeq RNA Sample Seq kit (Illumina Inc., USA) according to the manufacturer's protocol. Briefly, poly-A containing mRNA molecules were purified from 70 to 450 ng of total RNA using oligo-dT magnetic beads. The purified mRNA was fragmented by addition of the fragmentation buffer and was heated at 94°C in a thermocycler for 8 minutes. First strand cDNA was synthesized using random primers. Second strand cDNA synthesis, end repair, A-tailing, and adapter ligation was done in accordance with the manufacturer's instructions. Purified cDNA templates were enriched by 15 cycles of PCR for 10 seconds at 98°C, 30 seconds at 65°C, and 30 seconds at 72°C using Illumina's proprietary primers and Phusion DNA polymerase. Each indexed cDNA library was verified using a DNA 1000 Chip on a Bioanalyzer 2100 and quantified by real time PCR with the KAPA Library Quantification Kit for Illumina Sequencing Platforms (Kapa Biosystems Ltd, SA) then diluted to 10 nM using Illumina's resuspension buffer.

For each lane of sequencing, four libraries (ChIPseq) or 6 libraries (RNAseq) were pooled in equimolar concentrations, denatured using 0.1N NaOH and diluted to a final concentration of 7 pM (ChIPseq) or 8 pM (RNAseq) using Illumina's HT1 buffer. 120 μL of the dilution was then transferred into a 200 μL strip tube and placed on ice before loading onto the cBot. The flow cell was clustered using Single Read Cluster Generation Kit (Illumina Inc., USA), according to the Illumina SR_amplification_Linearization_ Blocking_PrimerHyb recipe. The flow cell was loaded onto the Illumina HiSeq 2000 instrument following the manufacturer's instructions and sequencing was performed with the 50 cycles, single read, indexed protocol. Image analyses and base calling were performed using the HiSeq Control Software (HCS) and Real-Time Analysis component (RTA). Demultiplexing was performed using CASAVA (Illumina). The quality of the data was assessed using fastqc from the Babraham Institute and the Illumina software SAV (Sequence Analysis Viewer).

### Illumina sequencing quality control, alignment and ChIP-Seq peak calling

All data treatment was carried out under a local galaxy instance [28](http://galaxy.univ-perp.fr). Fastq Groomer was used for verification of the fastqsanger format, and the FASTX-Toolkit (Compute quality statistics, Draw quality score boxplot, Draw nucleotides distribution chart) was used for initial quality control. Read quality was judged sufficiently good (the majority of reads showed a quality score ≥24 for all positions) and no further quality filter was applied.

For ChIP-Seq, reads were aligned to the *S*. *mansoni* reference genome, assembly version 5.0 (downloaded from ftp://ftp.sanger.ac.uk/pub/pathogens/Schistosoma/mansoni/genome/Assembly-v5/) using Bowtie2 [29] evoking parameters—sensitive-k 2. Mapping quality (mapq) in Bowtie2 is related to “uniqueness” of the read. The higher the quality score is, the lower is the probability that a read can map elsewhere in the genome. By filtering mapped reads with a score of 255 (the highest possible) in samtools [30] (samtools view-hS-q 255), we kept reads with a single match on the genome. In Bowtie 2.1 score 255 was abandoned and filtering of uniquely mapped reads was done with “samtools view-Sh-q *quality value 40–42*-F 0x0004 - | grep-v XS:i. “. SAM alignment files were converted into the bed format with pyicos [31] and sorted with sortBed-i of the bedtools suite [32]. For peak identification an equal number of random lines in the bed-file was chosen for each biological replicate (**Table 1**). Although comparative analysis software usually include a normalization step to handle the different numbers of mapped reads in conditions or replicates when comparing samples, we found that the random sampling step drastically reduces the amount of false positives without lowering the sensitivity. Mostly, this removes background peaks. Identification of peaks was done with ranger of Peakranger v1.16 [33] with P value cut off 0.0001, FDR cut off 0.01, Read extension length 200, Smoothing bandwidth 99 and Delta 1. We used the input samples as negative controls for the peakcalling (-c). For data visualization, wiggle files were generated and uploaded to a local GBrowse instance [34] (http://genome.univ-perp.fr).

**Table 1:** Summary of next-generation sequencing data and analysis results.

Condition	H3K4Me3			H3K9Ac		H3K9Me3		H3K27Me3		
	cercariae	adults	schistosomula	cercariae	adults	cercariae	adults	cercariae	adults	schistosomula
**Number of samples**	2	2	2	2	2	2	2	3	3	2
**Input reads (average per sample)**	34,519,715	6,421,317	49,109,097	54,833,141	43,884,060	53,702,142	43,884,060	40,895,894	33,865,527	46,158,137
**Alignment to unique sequences**										
Aligned exactly 1 time (average per sample)	21,455,086	3,097,077	28,275,831	28,626,785	28,035,327	23,326,061	21,945,541	22,882,234	27,123,469	30,651,658
Aligned reads used for peak-calling (per sample)	2,900,000	2,900,000	2,900,000	23,000,000	23,000,000	21,000,000	21,000,000	17,900,000	17,900,000	17,900,000
Regional peaks identified by peakranger 1.16 (average for condition)	7,746	8,868	7,376	17,116	421	128,518	100,884	145,322	27,544	65,629
Significantly different peaks identified by DiffBind	224			3,844		9,505		8,480		
DiffBind result confirmed by visual inspection	124			random sample of 100 peaks for each condition(33–50% confirmed by 2 independent observers)						
EpiChIP peaks with signal at FDR 0.01 (per condition)	3,598	3,566	3,954					4,801	4,370	5,024
**Alignment to repetitive sequences**										
Aligned exactly 1 time (average per sample)	1,326,620	242,000		3,805,162	3,091,945	3,881,193	3,612,100	3,999,829	4,554,097	
Random sample used for DESeq	220,000	220,000		2,900,000	2,900,000	3,400,000	3,400,000	3,900,000	3,900,000	

ChIP-Seq results in adults and cercariae were verified by qPCR (primers in **S1 Table**) in 20 randomly chosen regions using the following conditions: denaturation 95°C for 10 seconds, annealing 60°C for 10 seconds, extension 72°C for 20 seconds, over 40 cycles. The kit used for the amplification is the LightCycler 480 SYBR Green 1 Master (Roche, 04887352001) and the instrument is a LightCycler 480 (Roche). To compare ChIP-Seq and qPCR results, coverage was calculated with coverageBed of the bedtools suite for the regions whose boundaries were defined by the corresponding qPCR primer positions and divided by the coverage in the alpha-tubulin reference locus (Smp_scaff000423:21,107–21,289). Spearman rank correlation gave values > 0.8, R2 was 0.64 for H3K4me3 and 0.59 for H3K27me3 indicating that both methods gave comparable results (**S1 Fig**).

For RNA-Seq, reads were aligned to the genome with TopHat v1.4.1 with default parameters and exon-intron structure was reconstructed with cufflinks v1.3.0 evoking-q-I 40000-F 0.100000-j 0.150000-p 8 on each library and data were merged and linked to the gene prediction of the Sanger Centre (ftp://ftp.sanger.ac.uk/pub/pathogens/Schistosoma/mansoni/genome/Assembly-v5/) with Cuffmerge v1.0.0 [35].

### Comparative ChIP-Seq analysis

To identify peaks that were different between cercariae and adults, we used the DiffBind library 1.6.1 [36] available on Bioconductor [37] for R version 2.15.1. As input, we used the randomly sampled aligned reads that also served as inputs for PeakRanger and the regions file generated with PeakRanger, all in bed format. Diffbind allows for replicates and “input” negative control to be taken into account to search for differential peak distribution between conditions. For H3K9ac, PCR duplicates were removed with pyicos, which improved sample autocorrelation. For H3K4me3 all differences were inspected on GBrowse. For the other histone isoforms, one hundred regions identified by DiffBind were randomly sampled with a custom Perl script and verified by visual inspection. Average histone modification profiles around TSS were generated by doing a “window analysis” around the TSS of genes, using EpiChIP v0.9.7-e [38]. EpiChIP allows for generating genome-wide, average profiles for histone modifications at a given genomic feature (in our case, around the TSS for genes). For this purpose, we used the output file of Cuffmerge and selected only those 12,871 genes located on linkage groups that were assembled at the chromosome level. Chromosome names were converted in the input bed files into EpiChIP canonical names (“Chr*number*”) with a custom script. EpiChIP was also used to correlate transcription and histone modification, and to quantify the proportion of peaks that contribute significantly to the average profiles. Common peaks were detected from Peakranger region bed-files with multiIntersectBed of the BedTools package.

For the analysis of chromatin structure around repetitive sequences, reads were aligned to a fasta file containing all 3,145 known repeats of *S*. *mansoni* as consensus sequences (available http://methdb.univ-perp.fr/downloads/) [39] using Bowtie2 [29] evoking parameter–k 2 and parameter preset “sensitive”. Unique matches were filtered using samtools [30] (mapping quality mapq = 255). In order to obtain identical effective library sizes for each sample, the same number of aligned reads was randomly sampled for each of the ChIP experiments (**Table 1**). After the counts had been normalized, they were compared between samples using the DESeq package of the R software [40]. This package uses the negative binomial distribution to calculate the P-value for each element between two compared samples and find elements that present significant differences in their read enrichment levels.

### *In-situ* detection of replication and transcription

For the detection of mRNA synthesis, cercariae were incubated in spring water at 28°C with a concentration of 1mM of 5-ethynyl-uridine (EU, Invitrogen Click-iT RNA Imaging Kit, MP10329) for 2, 18 h and 21 h. Freshly transformed schistosomula were grown in Basch medium at the same EU concentration in an incubator at 37°C and 5% CO_2_, also for 2, 18, and 21 h. Labeling of replicating cells was performed in the exact same conditions, only substituting EU by a concentration of 10 mM of 5-ethynyl-2’-deoxyuridine (EDU, Invitrogen Click-iT DNA Imaging Kit, MP10337). Negative controls without the addition of 5-ethynyl-uridine (EU) and 5-ethynyl-2’-deoxyuridine (EDU) were also performed for every condition. Fixation, development and Hoechst 33342 coloration after incubation were performed following the protocols of the supplier. Imaging was done on a confocal microscope Zeiss LSM 700 at 488 nm (EU and EDU) and 405 nm (Hoechst 33342). For every condition, 100 animals were observed for transcription and replication.

### Accession numbers

RNA-Seq and ChIP-Seq reads are available at the NCBI-SRA under study accession numbers SRP034587 (ChIP-Seq) and SRP035609 (RNA-Seq).

## Results

### Chromatin landscape in *S*. *mansoni* cercariae and adults

**Fig 1:**
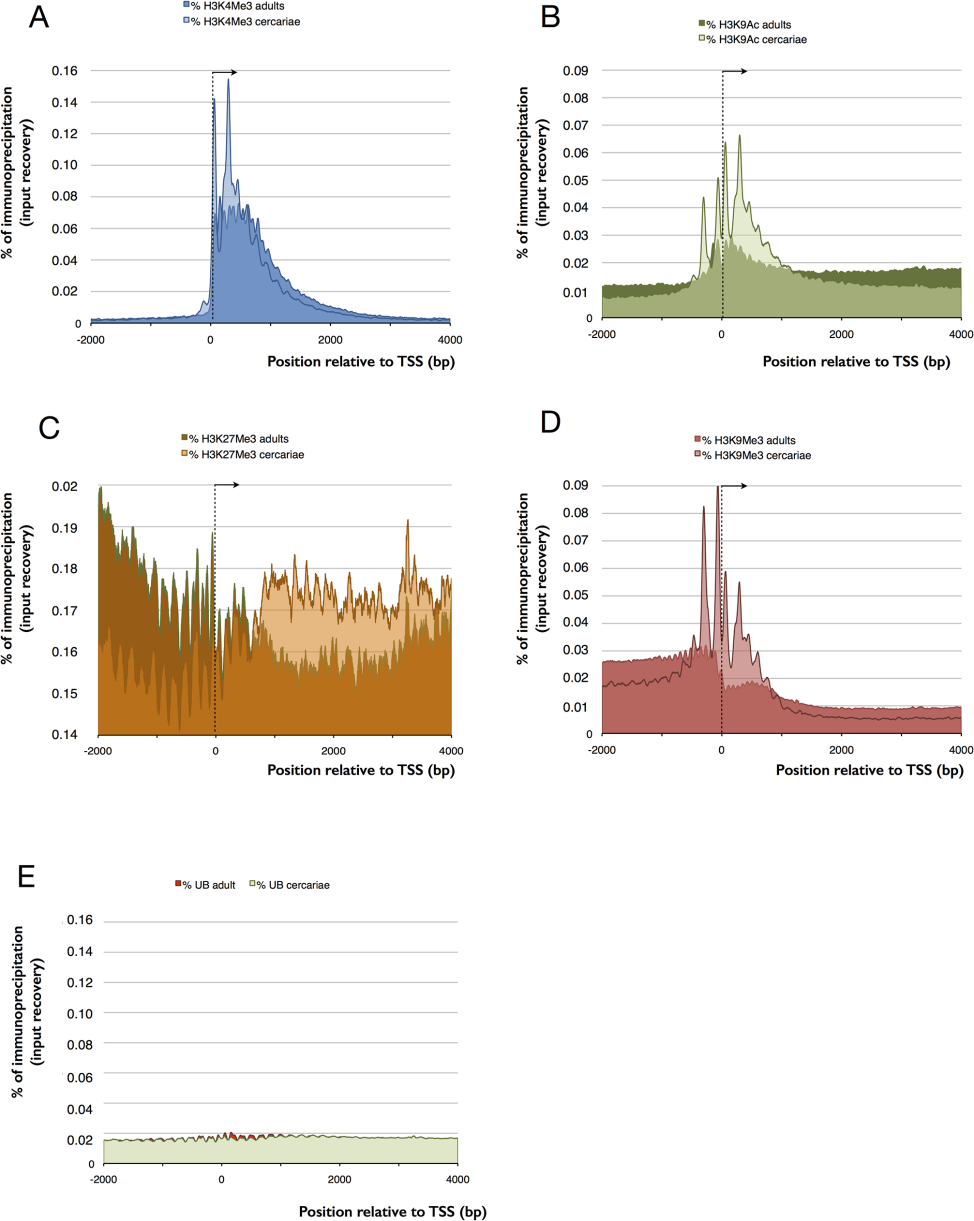
Comparison of EpiChIP profiles 2kb upstream and 4kb downstream of the TSS (average for 12,871 genes located on linkage groups that are assembled on chromosome level) for cercariae (light color) and adults (dark color). TSS and direction of transcription indicated by an arrowhead. X-axis in bp, y-axis in relative enrichment (% of all mapped reads). (A) H3K9me3, (B) H3K9ac, (C) H3K27me3, (D) H3K9me3, and (E) unbound fractions UB of mock ChIP without antibody that corresponds to input that shows no difference between cercariae and adults and no enrichment at the TSS.

**Fig 2:**
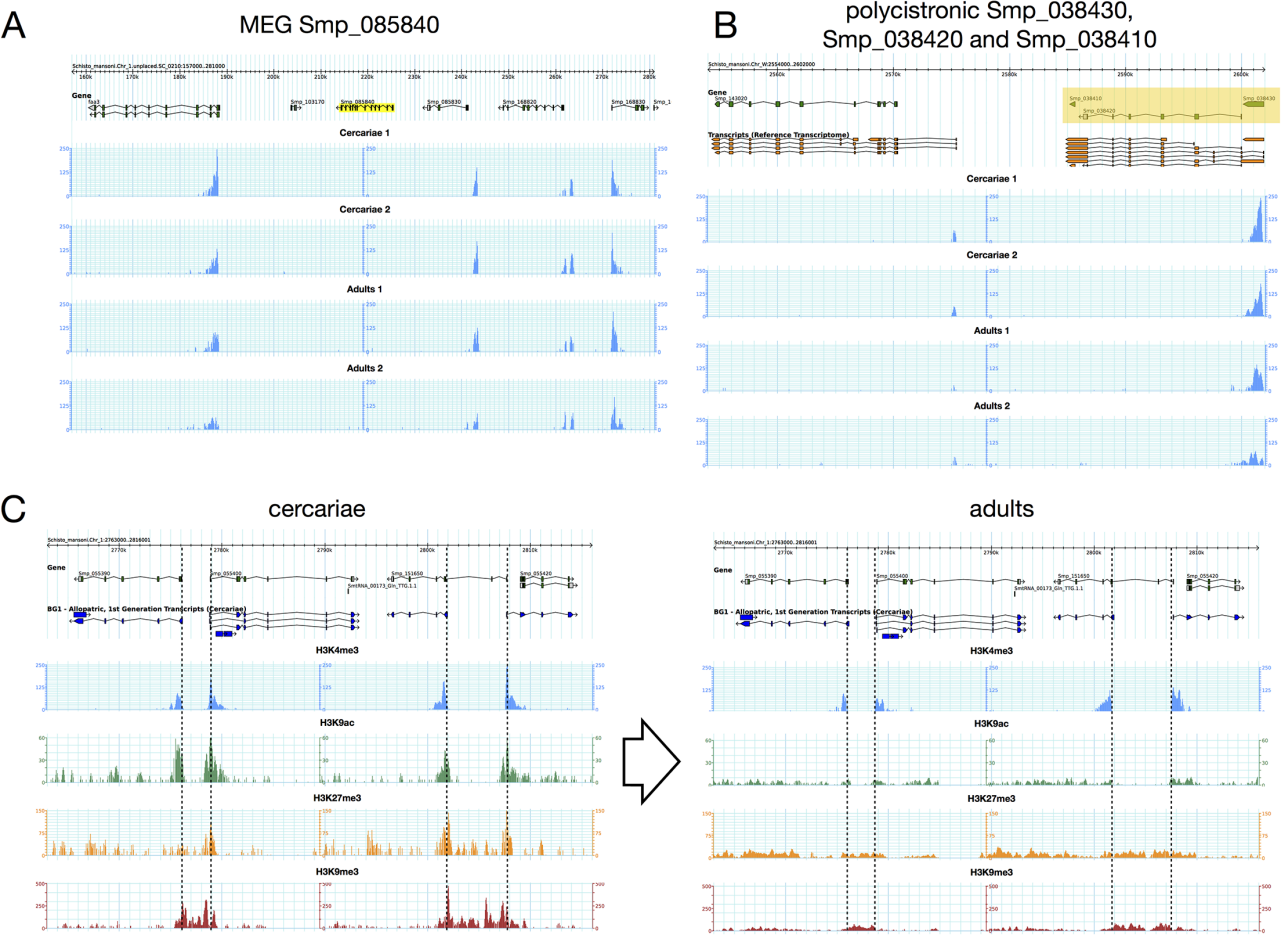
Typical examples of chromatin profiles. (A) H3K4me3 peaks are absent in the TSS of MEG Smp_085840 (highlighted in yellow) while surrounding genes show characteristic peaks in all 4 samples. (B) Polycistronic genes Smp_038430, Smp_038420 and Smp_038410 (from right to left, highlighted in yellow) show an H3K4me3 peak only in the first gene. (C) Changes in chromatin structure in cercariae (left) and adults (right). On top predicted genes, below RNA-Seq based exon/intron structure, followed by H3K4Me3 (blue), H3K9Ac (green), H3K27Me3 (yellow) and H3K9Me3 (red), identical scale for cercariae and adults. Positions of TSS that were detected by RNA-Seq are indicated by a dotted line.

To link transcription and chromatin structure, we considered the correct identification of the TSS as crucial. Anecdotal evidences had suggested that the available automatic annotation had missed a certain proportion of first exons and we decided to perform RNA-Seq with RNA extracted from cercariae. Indeed, our data showed that in about 20% of the genes, TSS had to be corrected. We then performed ChIP-Seq. For H3K4me3, we found relatively sharp peaks with a mean peak maximum located 250 bp (or 1–2 nucleosomes) downstream of the transcription start site (TSS) of genes (**Fig 1A**). We detected on average 7,746 peaks in cercariae and 8,868 peaks in adults (**Table 1**). For about ~24% of genes, EpiChIP identified transcripts but no significant H3K4me3 peaks (FDR = 0.01). Examples for genes that are free of H3K4me3 peaks are all known micro exon genes (MEGs) supplementary table S5.4 in [41] (**Fig 2A**), with the exception of Smp_163710 that shows small peaks in adults. MEGs contain unusually small exons of 3–36 bp that are repeated in tandem [42]. Their function remains elusive but it is thought that hypervariable proteins can be generated by MEGs through a ‘pick and mix’ splicing strategy. It is conceivable that such types of genes have a specific control mechanism. Other H3K4me3-peak free genes are polycistronic genes that are located downstream of the trans-splicing site. Protasio *et al*. estimated trans-splicing events to occur in 11% of *S*. *mansoni* genes [18] and had compiled a list of putative polycistronic transcripts. We used this list and show that in 143 (89.9%) genes, the H3K4me3 peak is located exclusively in the gene upstream of the trans-splicing acceptor site (**Fig 2B**), in 2 (1.2%) cases peaks are present in both genes (upstream and downstream), and in 13 (8.2%) genes we did not find any peak, although in 3 cases we suspect that an upstream gene with a peak may be the first of the polycistronic unit (**S2 Table**). Our data are in line with the view that these genes are indeed transcribed as one unit from a single TSS (that has the H3K4me3 signal) and fragmented by trans-splicing. For H3K27me3 we found with Diagenode antibody cat# pAb-069-050 lot# A29900242 enrichment at the TSS (-500 to +1,000 bp), while it was reduced to background level in adults. Since there was anecdotic evidence for cross-reactivity of this antibody with H3K4me3, we repeated the experiment with cat# C15410069 (that replaces pAb-069-050) lot# A1821D. With this antibody we also observe enrichment of H3K27me3 in cercariae compared to adults, but shifted towards the body of the gene (**Fig 1C** and **Fig 2C**). Methylation and acetylation in H3K9 are mutually exclusive at the same histone and H3K9me3 is considered a repressive mark. H3K9me3 shows a peak about 200 bp upstream of the TSS in cercariae, which is reduced to an upstream background in adults (**Fig 1D** and **Fig 2C**). H3K9ac is a hallmark of active transcription. In cercariae we detected this modification primarily around the TSS (-500 to +1,000 bp) while in adults acetylation spans the body of the genes (**Fig 1B** and **Fig 2C**). Taken together, our results indicate that all four studied histone modifications show an unusual enrichment around the TSS in cercariae, while in adults their distribution is similar to that found in other metazoans. We saw no differences between the input fractions (negative controls used for normalization) of cercariae and adults (**Fig 1E**).

**Fig 3:**
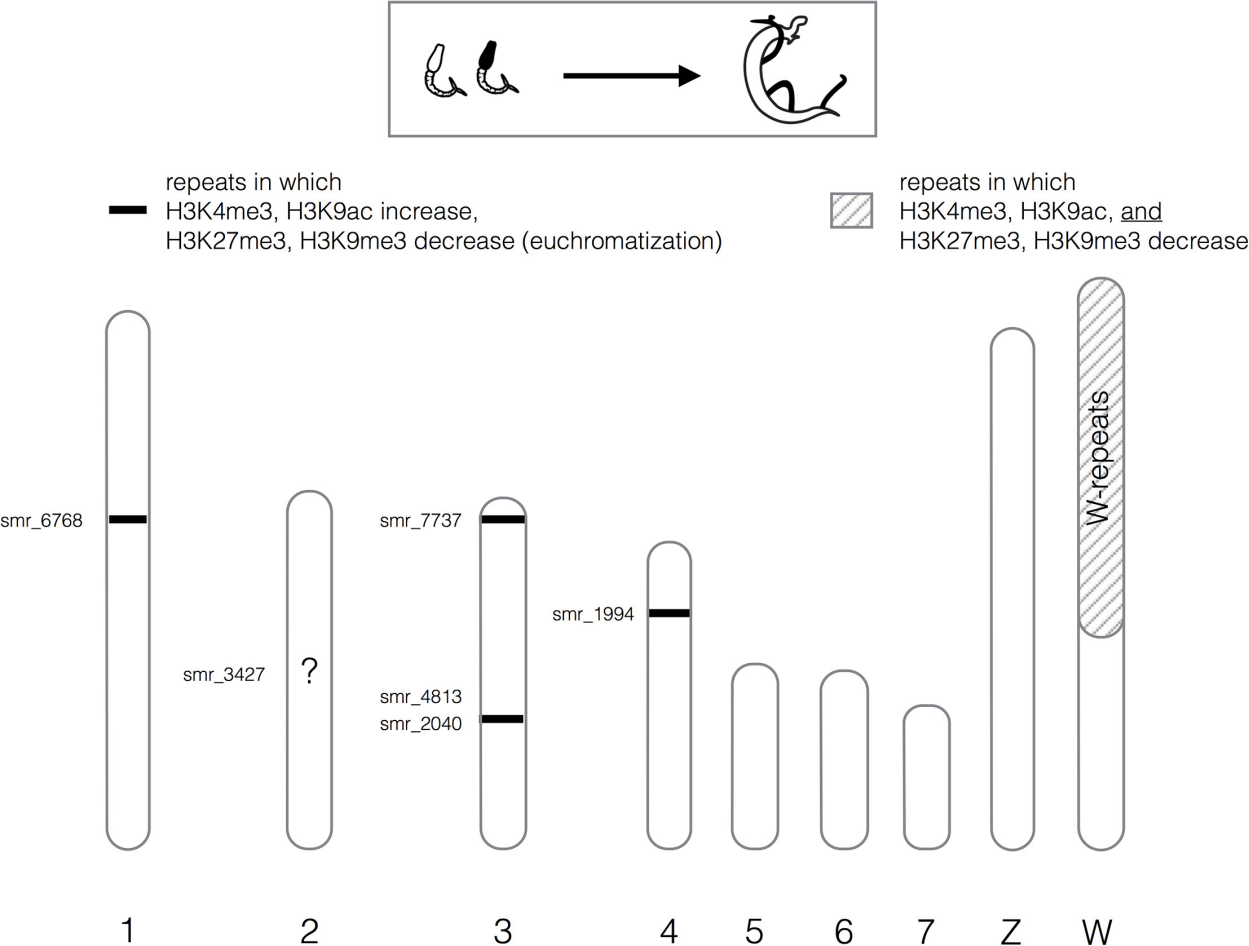
Schematic representation of chromatin structure changes in tandem-repeat regions during metamorphosis of cercariae into adults. Autosomes 1–7 are labeled with numbers, sex chromosomes with letters. Repeat regions are indicated on each chromosome as inferred by alignment to the reference genome, or previously by FISH on the W-chromosome. Repeat class smr_3427 is presumably on chromosome 2 but the exact position is unknown (unpositioned contig).

In cercariae and adults, both H3K4me3 and H3K9ac showed globally low association with repeats (4.10±0.43% and 6.45±0.65%, respectively of the total reads aligned) (details in **Table 1**). For H3K27me3 and H3K9me3 the percentage was higher with 7.60±1.00% and 7.75±0.60%. DESeq [40] identified repeats that change chromatin structure during transformation from cercariae to adults. DESeq uses a negative binomial distribution to model read counts that can be assigned to a locus. To estimate variance within a sample it pools the data from genes with similar hit counts, thus allowing variance estimation and identification of significant differences based on a small number of biological replicates [40]. We used DESeq because we had earlier compared it to edgeR [43], and DESeq2 and in our hands it gave the most consistent results. Using a p-value of < 10^−5^ as the selection criterion, we identified 17 (0.54%) repeats that change their chromatin status for H3K4me3, 182 (5.79%) for H3K9ac, 263 (8.36%) for H3K9me3, and 40 (1.27%) for H3K27me3. Results are summarized in **S3 Table**. Interestingly, not all repeats were concerned equally: in roughly two thirds of these repeats (n = 363, 11.5%) more than one histone isoform had changed meaning that about 10% of all repeat families undergo important chromatin structure changes during the transformation of cercariae into adults. Two results were particularly interesting (**Fig 3**). The first is a chromatin change in five regions that are composed of tandemly repeated stretches of class I LTR retrotransposons and class II DNA transposons. These regions are located on the large *S*. *mansoni* chromosomes (1 to 4), but there might be similar regions on the three smaller chromosomes that were overlooked. Each of the regions is composed of a different repeat, but they all belong to the same repeat class (*i*.*e*. transposon). In these five regions, enrichment of histone forms H3K9me3 and H3K27me3 decreases during cercariae to adult metamorphosis, while enrichment of H3K9ac and H3K4me3 increases. Considering five states (H3K4me3 up, H3K9ac up, H3K9me3 down, H3K27me3 down, transposon or other), the individual term binomial distribution probability to obtain such a combination by chance is 3.7^−36^. Our data suggest therefore that the process is not randomly distributed along the genome. Stretches of (retro)transposons could form heterochromatic knobs in the cercariae and their change in chromatin structure could be an indication of large scale chromatin rearrangements or chromosome displacements in the nucleus during transformation into adults. Another peculiar observation during transformation from cercariae to adults is the nearly complete loss of H3 modifications in 22 of the 36 W-specific repeats (61%). We had shown earlier that transcription of these repeats can only be observed during larval stages and is undetectable in adults [44]. From qPCR data for a small subset we had already postulated that these repeats acquire an “adult-specific” chromatin structure. This view is confirmed by the new NGS data: H3K9/K4/K27 methylation and H3K9 acetylation are lost. Cytogenetic images suggest that the repeat containing large pericentromeric regions becomes heterochromatic when cercariae develop in the snail, but no clear data is available for adult worms [45]. It is tempting to speculate that H3 is replaced by another H3 isoform similar to what is observed in centromeres and neo-centromeres of other species for instance [46]. A homologue of the centromere specific form of H3, CENP-A, was detected in *S*. *mansoni* (Smp_040020) [47] but homology with CENP-A of other species is vague and basically restricted to the core H3/H4 domain.

### Bivalent trimethylation of H3K4 and H3K27 is specific for a subset of genes in cercariae

**Fig 4:**
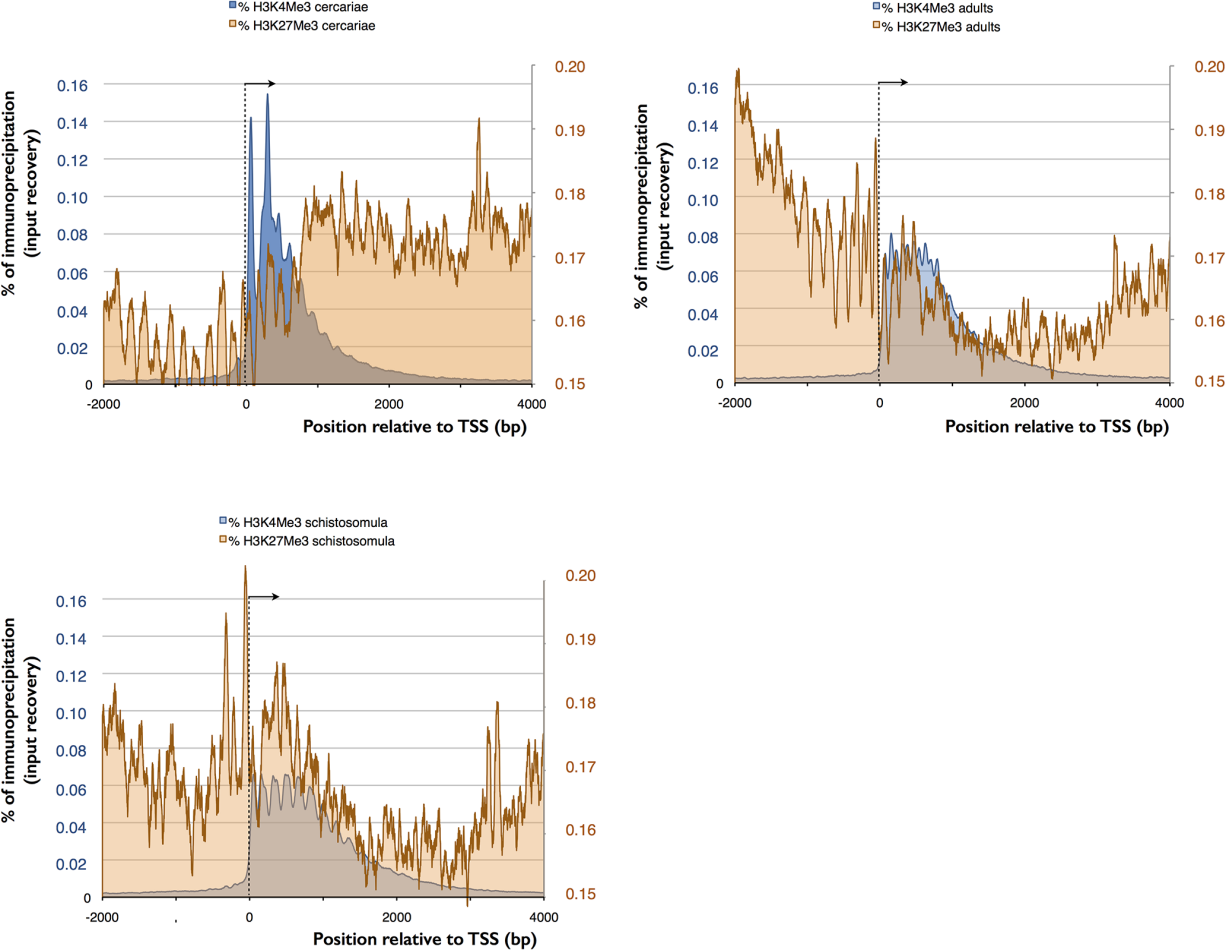
Comparison of EpiChIP profiles 2 kb upstream and 4 kb downstream of the TSS (average for 12,871 genes located on linkage groups that are assembled on chromosome level) between different histone isoforms in cercariae (left up), schistosomula (left down) and adults (right). TSS and direction of transcription indicated by an arrowhead. Bivalent methylation of H3K4 and H3K27 occurs in cercariae but not in adults, left y-axis for H3K4me3, right y-axis for H3K27me3.

While PeakRanger identify 1,122 more peaks in adults than cercariae for H3K4me3, Diffbind considers only 224 differences to be statistically significant. After visual inspection, we confirmed differential enrichment in only 124 regions along the genome with 95 (77%) hypermethylated in cercariae and 29 (23%) in adults. This means that less than 2% of H3K4me3 peaks are different, while gene transcription patterns of cercariae and adults are clearly distinct [48]. A peculiar feature of the *S*. *mansoni* epigenome was revealed when we analyzed the distribution of the heterochromatic H3K27me3. In cercariae, H3K27 trimethylation is high in the gene body (**Fig 4**). H3K27me3 and H3K4me3 are also statistically significantly enriched around the TSS in 150 genes. This number was reduced to 121 after visual inspection (**S4 Table**). Thirty-five days of development separate cercariae and full-grown adults worms. To determine more precisely the time window during which chromatin is remodeled we performed ChIP-Seq on schistomula. Our results show that the adult chromatin structure is already established in schistosomula by 18 h after infection (**Fig 4**). Most importantly, in schistosomula, bivalent methylation is absent in the 121 genes that were significantly enriched in cercariae.

### Cercariae are transcriptionally silent

**Fig 5:**
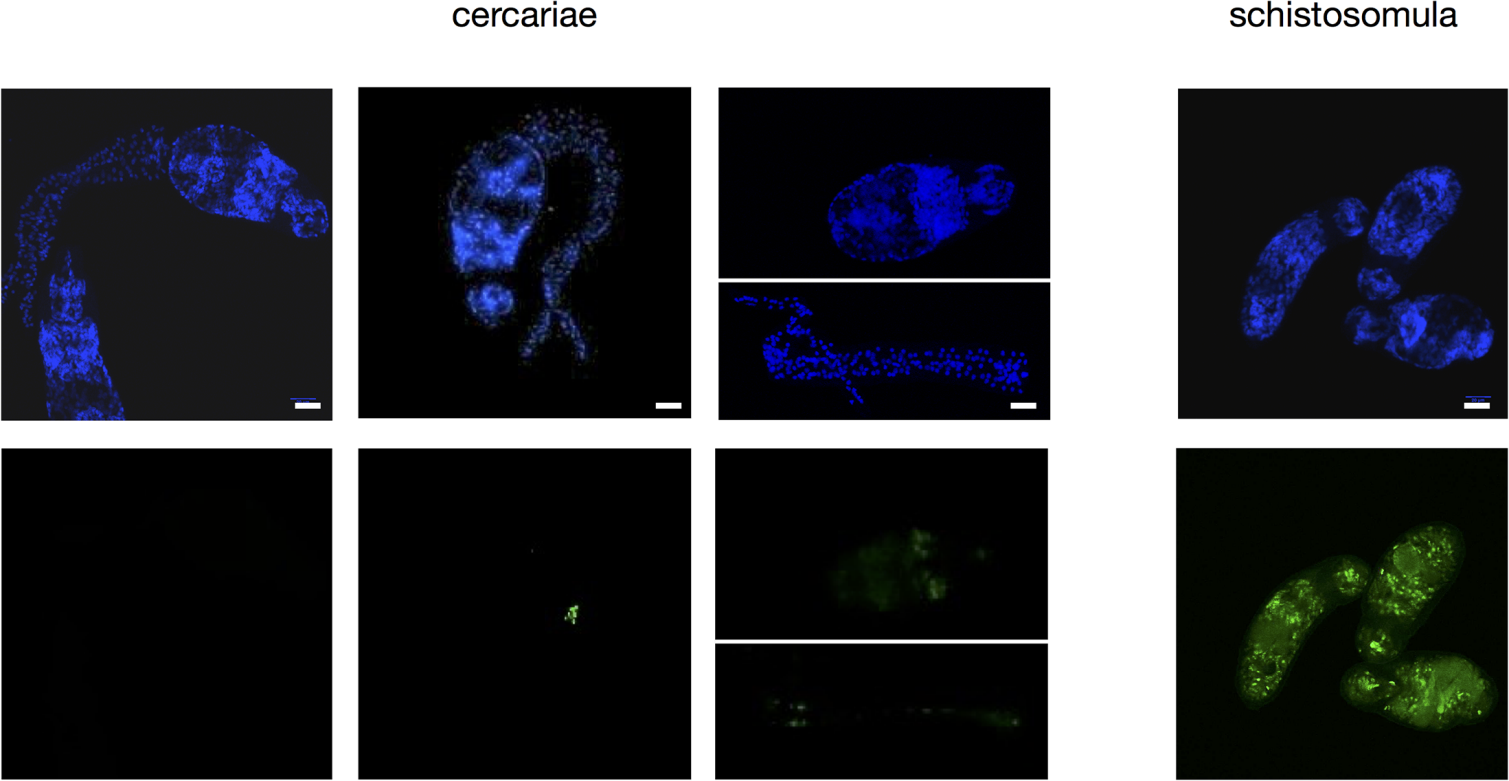
Detection of RNA synthesis by incorporation of EU by fluorescence microscopy. Top panel (blue) Hoechst 33342 staining of DNA, bottom panel (green) EU incorporation and detection with Alexa Fluor 488. White bar 20 μm.

**Fig 6:**
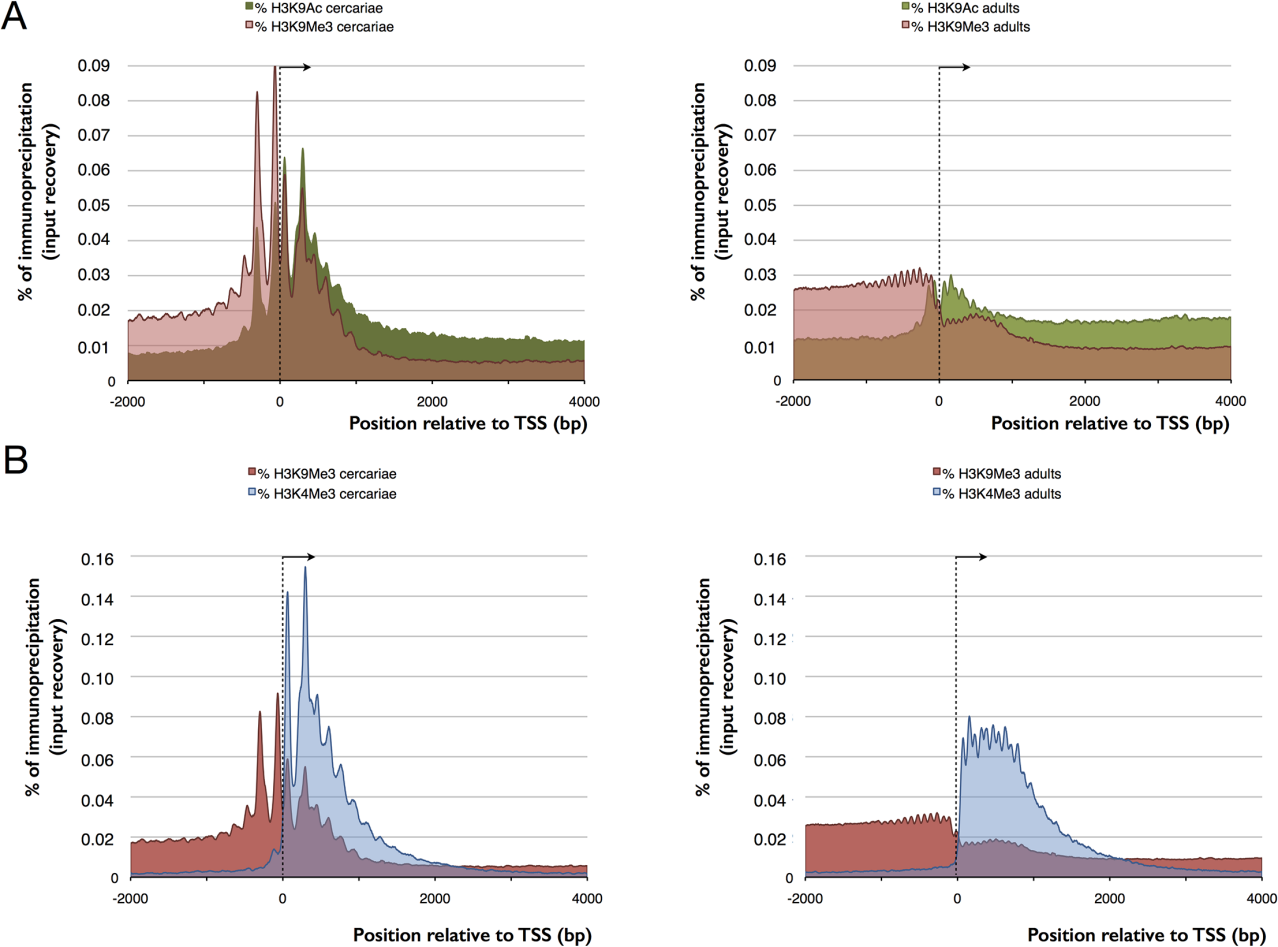
Comparison of EpiChIP profiles 2 kb upstream and 4 kb downstream of the TSS (average for 12,871 genes located on linkage groups that are assembled on chromosome level) between different histone isoforms in cercariae (left) and adults (right). TSS and direction of transcription indicated by an arrowhead. (A) methylation of H3K9 upstream of TSS and acetylation downstream, concentrated around TSS in cercariae and spanning the gene body (H3K9ac) and the intergenic region (H3K9me3) in adults, (B) methylation of H3K9 and H3K4 are mutually exclusive in adults but overlap in cercariae.

While RNA is clearly present in cercariae, the co-localization of all four histone modifications at the TSS made us wonder if transcription is indeed ongoing or if it is poised. Cercariae are short lived and they do not grow outside the host. It is therefore conceivable that their RNA was produced during their development in the snail. We used incorporation of the cytosine analog 5-ethynyl-uridine (EU) and fluorescence labeling to detect active transcription *in vivo*. At 28°C, the maximum life span of a cercaria is 32 h (90% of mortality occurs past 26 h [49]) but infection capacity decreases rapidly and probably does not exceed 6 h. We decided therefore to use a relatively long EU pulse of 18 h. Despite this, we detected fluorescence signals in less than 10% of the cercariae. With the exception of a single individual (out of one hundred) signals were detected in only a few of the roughly 1,000 cells of an individual larva, and were always restricted to the nucleus (examples shown in **Fig 5**). In contrast, schistosomula, the first developmental stage after infection of the vertebrate host, showed consistent staining in the 100 arbitrarily chosen individuals and staining was more dispersed. We conclude that there is very little transcription in cercariae and that transcription is rapidly activated upon transformation into schistosomula. Our observation that in cercariae the repressive mark H3K9me3 is slightly upstream and the permissive mark H3K9ac is slightly downstream but close to the TSS is in accordance with stalled transcription. In contrast, in adults H3K9 acetylation and methylation are spread out with H3K9ac occurring along the gene body and H3K9me3 in the upstream regions separating genes (**Fig 6A**).

Bivalent H3K4/H3K27 trimethylation and absence of transcription are reminiscent of what occurs in ESC of vertebrates, *e*.*g*. mice [13], human [50] and zebrafish [51]. There, bivalent H3K4/H3K27 trimethylation occurs at the transcription start site of lineage-specific genes and it was proposed that it keeps them transcriptionally “poised”, with the transcription machinery in place, ready to start transcription upon removal of the H3K27me3 mark (reviewed in [51]). ESC do not exist in schistosomes, but totipotent somatic stem cells (neoblasts) were found in platyhelminthes from the free-living turbellarians (for review [52]) to the endo- or ecto-parasitic flatworms (monogenea, cestoda, trematoda) [53]. Recently, somatic stem cells were detected in adult schistosomes and called proliferating somatic cells (PSCs) [23,54]. Another type of stem cells, totipotent germinal cells, is present in the intramolluscan stages of *S*. *mansoni* but they are restricted to a small germinal cell cluster in cercariae [23]. Chromatin structure profiles for these cells have not been studied but it could be that cercariae possess simply a large number of ESC-like cells. The ultrastructure of cercariae was described in great detail in the past [55,56] and apart from the above-mentioned small population of germinal cells, no stem cells were detected. Stem cells could be identified thanks to the expression of characteristic marker genes such as vasa, piwi, tudor and nanos. However, piwi and tudor-domain containing genes are absent from flukes and tapeworms and no true vasa orthologue has been identified in schistosomes [41]. Thus, we sought to exploit rapid replication as another characteristic feature of these cells to identify them in schistosomes.

### No DNA replication in cercariae

We used 5-ethynyl-2’-deoxyuridine (EDU) incorporation to detect replication in cercariae and schistosomula. While the latter showed at least one replicating cell per individual in 27% (36 out of 135) after 2 h EDU pulse and 86% (156 out of 181) after 18 h, in none of the cercariae did we detect replication. Consequently, cercariae do not contain large amounts of rapidly replicating ES-like cells or neoblasts that could explain the ChIP-Seq profiles.

## Discussion

We describe here the epigenome of *S*. *mansoni* using four major histone modifications. We analyzed unique sequences, in particular those containing genes, but also the repetitive compartment of the genome.

The enrichment of histone forms around TSS shares similarities with other metazoans, but also shows particularities. Similar to what is found in model organisms, H3K4me3 is enriched at transcription start sites (TSS) and promoter regions of genes with actual or potential transcription [2,5,57–59]. In mammalian genomes, it marks active or poised (when combined with a repressive mark) high CpG-content promoters, while is only present at low CpG-content promoters when the latter are active. Further analysis will show if this is also the case in schistosomes and for what reason some TSS are devoid of H3K4me3. H3K9ac is another mark associated with euchromatin and facilitation of transcription [2–6]. The main enrichment of this mark is often observed between the beginning of promoters and the 5’ end of genes, and sometimes in enhancers [2,3,7]. In *S*. *mansoni* there is a clear difference between cercariae and adults with H3K9ac spanning the gene body only in the latter. H3K9me3 is a landmark of constitutive heterochromatin. In many organisms it is highly enriched at telomeric, pericentromeric and repeat-rich regions, and is responsible for stable large-scale repression [8–11]. In humans, it was shown that this modification is mutually exclusive with H3K4me3 [12]. This is also the case in adult schistosomes (**Fig 6B**). Nevertheless, H3K9me3 can be present at a very high level over transcribed regions of active mammalian genes, especially 1.5 kb after the TSS, and at a low levels at the promoter [60]. H3K9me3 increases during activation of transcription and is quickly removed afterwards [60]. In our study, association of H3K9me3 with the TSS is only observed in cercariae, where this mark is predominately located upstream of the gene. In *S*. *mansoni*, a combination of H3K9me3 and H3K9ac allows the prediction of the direction of transcription (always from the H3K9me3 peak to the H3K9ac peak) and it seems that blocks of H3K9me3 separate promoters of genes that are transcribed back-to-back (example shown in **Fig 2C**). This might be a signature of protein-coding genes. The fourth studied histone isoform, H3K27me3 is known to maintain facultative heterochromatin and is involved in the regulation and sequential expression/repression of developmental genes [11,61]. It is present at inactive or poised (when combined with H3K4me3) high CpG-content promoters and is anti-correlated with the level of transcription [9,10]. In some unicellular organisms, such as *Plasmodium falciparum* this mark can even be absent [6]. In *S*. *mansoni*, the mark is weak in genes of schistosomula and adults, but present in cercariae. Similar to ESC, bivalent trimethylation of H3K4 and H3K27 is present in at least 121 genes. Bivalent methylation is often considered as an ESC specific mark that leads to “poised” transcription that can rapidly be activated when the cells enter into differentiation. Our data indicate that there are no rapidly proliferating stem cells in cercariae, and we show that there is no transcriptional activity. Consequently, neoblasts of cercariae are either in a dormant state, or somatic cells of cercariae use the same mechanism as ESC to poise transcription that is rapidly activated after infection, upon transformation into schistosomula (at least during the first 18 h after penetration in the host). Bivalent H3K4/H3K27 trimethylation could therefore be a phylogenetically very old and/or more general mechanism to poise transcription than currently thought. However, as only 121 genes were detected to have these bivalent marks, another mechanisms could be involved in the transcription silencing observed in cercariae. At the moment, we can only hypothesize that the major shift of H3K27me3 enrichment (from gene body in cercariae to upstream of TSS in schistosomula and adults, **Fig 4**) and/or the strong enrichment of H3K9me3 and H3K9ac at the TSS of cercariae play a role in this phenomenon.

While the importance of repetitive sequences for genome structure and evolution is generally accepted, they are often considered too difficult to analyze by genome-wide approaches. Almost half of the *S*. *mansoni* genome is composed of repeats [39] and we deemed it to be important to include these in our analysis. Here we used a straightforward approach to evaluate chromatin structure in all known repeat classes of *S*. *mansoni*. We show that in five mini-satellite regions composed of retrotransposons H3K4me3 and H3K9ac increase while H3K9me3 and H3K27me3 decrease during the transition from cercariae into adults. It is difficult to evaluate the functional importance of this chromatin structure change. It was shown in cultured human cells that another type of satellite DNA (gamma satellites) is enriched for H3K4me3, and that it shields adjacent genes from heterochromatisation [62]. Acetylation of H3K9 was not investigated in this latter study. A similar effect might occur in our system but further work will be necessary to elucidate the function of these repeat regions.

In summary, we present here the epigenomes of *S*. *mansoni* for three life cycle stages. We show that in cercariae, bivalent H3K4/H3K27 trimethylation exists. To the best of our knowledge, this bivalent trimethylation has, in the animal kingdom, so far only been found in vertebrate ESCs. We also show that TSS occupancy by H3K4me3 is stable between cercariae and adults in the large majority of genes. It changes drastically for H3K9 trimethylation and acetylation. In addition, we identified at least five repeat locations that gain euchromatic status during development into adults. Our findings support a model in which transcription is poised in cercariae due to the presence of both activating and repressive marks. It is reactivated, presumably through the removal of the repressive methylation at the TSS and in the gene body, and in conjunction with large scale chromatin remodeling (indicated by changes in repeat regions). In this model, histone-modifying enzymes, in particular H3K27 and H3K9 demethylases would act within 18 h after skin penetration to release the poised state and allow for rapid activation of the transcription machinery that is already in place around the TSS. We had shown before that histone-modifying enzymes are indeed essential for transformation and infection success of another life cycle stage, the miracidia [63]. Others have shown that inhibition of HDAC8 leads to parasite death [64]. In zebrafish, a H3K27 demethylase of the KDM6 type (UTX) is needed for fin regeneration [65]. An orthologue of UTX is encoded in the *S*. *mansoni* genome (Smp_034000) and by q-RT-PCR we found that mRNA for this gene is present in cercariae, all four stages of schistosomula *in vivo*, and in male and female adults. Further studies will show if blocking this candidate will result in developmental arrest. Besides being of academic interest, such enzymes would also be promising new drug candidates, especially in the light of the fact that the only drug currently available to cure schistosomiasis, praziquantel, is ineffective against the larval forms [66]. In addition, many parasites possess infective life cycle stages that develop immediately after infection. Our findings should motivate search for characteristic histone modifications in these stages.

## Supporting Information

S1 Table (a) Primers targeting regions with bivalent H3K4me3 and H3K27me3 marks in cercariae, and monovalent H3K4me3 marks in adults. (b) Primers targeting regions with H3K4me3 differences between cercariae and adults. (DOCX)

S2 Table H3K4me3 peaks in putative polycistrons with a maximum intergenic distance of 200bp. Upstream indicates the transcript that is located upstream of the trans-splicing acceptor site. Down-stream indicates the putative trans-spliced transcript. Polycistron list from http://www.plosntds.org/article/info%3Adoi%2F10.1371%2Fjournal.pntd.0001455#s5 (S2 Table).
(XLS)

S3 Table DESeq results for ChIP-Seq on repetitive sequences. (XLSX)

S4 Table List of transcripts for which TSS has H3K4me3 and H3K27me in cercariae, evidence from GeneDB (for Smp_######) and from AmiGO v.1.8 (for TCONS_########). (PDF)

S1 Fig Relative enrichment compared to the alpha-tubulin locus: ChIP-Seq vs qPCR, p-value is given for the t-test of the null hypothesis that the corresponding slope is equal to zero against the two-sided alternative. (TIFF)
