# Supplementary material for: The Epigenome of Schistosoma mansoni Provides Insight about How Cercariae Poise Transcription until Infection
Source: PLoS Negl Trop Dis. 2015 Aug 25;9(8):e0003853. doi: 10.1371/journal.pntd.0003853 (PMC4549315; doi:10.1371/journal.pntd.0003853)
Supplement: S1 Table — (DOCX) [file pntd.0003853.s001.docx]

| **Supplementary table 1:**  **(a) Primers targeting regions with bivalent H3K4me3 and H3K27me3 marks in cercaria, and monovalent H3K4me3 marks in adults** | | |
| --- | --- | --- |
| ***Name*** | ***Forward sequence*** | ***Reverse sequence*** |
| sm.Chr_1:47971550,47972100 | gcacgtaaaccaccagcccca | caagcgaaaatccacgcgcca |
| sm.SC_0034:2246640,2247240 | gctcgcggagagtaaacccga | acgccgcctgaagatcaaggg |
| sm.SC_0071:1183830,1184320 | tgaccagtgtggaaatgacctgttgt | agtgaccactcgataggcacgca |
| sm.Chr_1:8165260,8166110 | gacctgggtgcactgggcaa | tggacgaaagtctcgttcttgatctct |
| sm.Chr_2:21540210,21540530 | tgctttgtgtgatttccaaagcccatt | agatcacagcccgagccttct |
| sm.SC_0225:87490,88050 | tgtccagcaaaatcccatgtcgc | tgtgttgcttgttgtccacagga |
| sm.Chr_2:26997540,26998000 | tcgtctcgcaatttacttttgagcca | caatgactgtgttgcggaatatctggt |
| sm.Chr_3:12856600,12856890 | tcctccacccccacgtacgttat | ccggtttgagttgccatacccca |
| sm.Chr_1:40471080,40471790 | tggtttagcttacgtgcgagagttc | acgcgcttgtgagtgtgactgt |
| sm.Chr_W:44511380,44511690 | tgaggcgagataggacacattcattca | agatggtcattaggaacagccgaact |
|  |  |  |
|  |  |  |
| **(b) Primers targeting regions with H3K4me3 differences between cercaria and adults** | | |
| ***Name*** | ***Forward sequence*** | ***Reverse sequence*** |
| XLOC_002337 | ccgtcacggccaataagggcg | gctcccatatgatgctcgattggct |
| XLOC_004599 | tgagcgagagtgatgctcttttcg | acagaagctgacgcccctga |
| XLOC_004718 | aagtggacgagaccggccca | aggttgcgaaaagccgacgaca |
| XLOC_010397 | tgggtggtggtttgcttgtgga | cgccccaaaactcgcaagcc |
| XLOC_004811 | gcctgacgctagcgactgaaca | tccggacaggttggggtgtct |
| XLOC_011635 | agaaccactgagggttatggtgtgt | ttcgctcctccagcccacaa |
| XLOC_001136 | gggcggcagaatcagggtcg | gggaactccggagacccaca |
| XLOC_006515 | tccacccactttcaccgaccact | acgtcagctgtcgctactacca |
| XLOC_006717 | gctactcaacatatcctgatctcccgt | gattgagctctattttgaggaagccga |
| XLOC_001871 | ccgatcgtgcgcttcgtcgat | agccttgatgccagctttccga |
