# Supplementary material for: The Epigenome of Schistosoma mansoni Provides Insight about How Cercariae Poise Transcription until Infection
Source: PLoS Negl Trop Dis. 2015 Aug 25;9(8):e0003853. doi: 10.1371/journal.pntd.0003853 (PMC4549315; doi:10.1371/journal.pntd.0003853)
Supplement: S4 Table — (PDF) [file pntd.0003853.s004.pdf]

Supplementary Table 4: List of transcripts for which TSS has H3K4me3 and H3K27me in cercariae, evidence from GeneDB (for Smp_######) and from AmiGO v.1.8 (for TCONS_########)

Transcript	Putative Function
TCONS_00009655	peptide chain release factor 1
TCONS_00013757	telomerase associated protein 1
TCONS_00004337	component of oligomeric golgi complex 5
TCONS_00026171	B-cell CLL/lymphoma 2b
Smp_078560	hexokinase
TCONS_00016671	ALG2, alpha-1,3/1,6-mannosyltransferase
Smp_043030	hexokinase
TCONS_00023960	hypothetical protein
TCONS_00016240	Transducin-like enhancer protein 4
Smp_032280	RNA binding protein MEX3B
Smp_059790	transketolase
Smp_152300	hypothetical protein
Smp_196600	BTB:POZ domain containing protein KCTD3
Smp_060270	protein jagunal
Smp_049160	zinc finger protein
TCONS_00112540	hypothetical protein
Smp_033950	Smad family member 4
Smp_194530	adenylate kinase isoenzyme 5
SmtRNA_00576_Pseudo_TTG.1.1	tRNA
Smp_147400	sestrin 1
Smp_158910	cyclin dependent protein kinase CDC28 regulatory
SmtRNA_01343_Pseudo_AAG.1.1	tRNA
TCONS_00003597	actomyosin contractile ring
Smp_130270	u11:u12 small nuclear ribonucleoprotein 48 kda
Smp_129500	dna directed rna polymerase i largest
TCONS_00006552	Monocarboxylate transporter 1
Smp_197420	hypothetical protein
Smp_054800	glycerophosphocholine phosphodiesterase GPCPD1
Smp_063110	Zinc finger MYM type protein 3
Smp_162540	tektin 3
Smp_169360	kinesin protein kif26a
Smp_030250	NF-YA subunit
Smp_003990	triosephosphate isomerase 1a
Smp_091460	glutamine synthetase
Smp_082780	wiskott Aldrich syndrome protein
TCONS_00011595	hypothetical protein
Smp_152310	hypothetical protein
TCONS_00011520	MORN repeat containing 3
Smp_036590	Regulator of differentiation 1
Smp_055420	transmembrane protein 222
Smp_060160	sperm flagellar protein 1
Smp_049250	major egg antigen
Smp_017990	hypothetical protein
Smp_040390	transcription factor Sp8
Smp_008260	glycogen synthase kinase 3 alpha
Smp_065110	LETM1 and EF hand domain containing protein 1
Smp_001560	e3 ubiquitin protein ligase ZSWIM2
Smp_071520	hypothetical protein
Smp_003700	28s ribosomal protein s18a mitochondrial
Smp_035360	nucleolar protein 14
Smp_054440	tensin
TCONS_00020090	integrin, alpha 7
Smp_200670	hypothetical protein
Smp_045530	hypothetical protein
Smp_212340	hypothetical protein
TCONS_00020393	Uncharacterized protein
Smp_092320	RNA polymerase II elongation factor ELL
TCONS_00014948	Uncharacterized protein
TCONS_00011880	Myosin light chain kinase, smooth muscle
TCONS_00030975	dolichol kinase
SmtRNA_00277_Pseudo_TTG.1.1	tRNA
Smp_211090	peptide chain release factor 1
Smp_053640	hypothetical protein
TCONS_00003055	N(alpha)-acetyltransferase 30, NatC catalytic subunit
Smp_127690	hypothetical protein
Smp_058150	NADH dehydrogenase (ubiquinone) 1 beta
Smp_027000	abhydrolase domain containing protein FAM108C1
TCONS_00000151	Succinate dehydrogenase [ubiquinone] iron-sulfur subunit, mitochondrial
Smp_125220	Aquaporin 9 (AQP 9) (Small solute channel 1)
TCONS_00011520	MORN repeat containing 3
Smp_042160	fructose-bisphosphate aldolase
TCONS_00027110	forkhead box J1b
Smp_137170	plasma membrane calcium transporting ATPase
Smp_072370	RNA polymerase associated protein CTR9
SmtRNA_00350_Pseudo_TTG.1.1	tRNA
Smp_115290	Ran specific GTPase activating protein
Smp_169970	kinetochore protein ndc80
Smp_134560	serine:threonine protein kinase doa
Smp_053040	charged multivesicular body protein 6
Smp_006060	hypothetical protein
TCONS_00004975	Bromodomain adjacent to zinc finger domain protein 2B
Smp_021160	peptidyl prolyl cis trans isomerase 2
Smp_179660	hypothetical protein
Smp_053550	hypothetical protein
TCONS_00015249	hypothetical protein
Smp_202560	hypothetical protein
Smp_089870	histone H2A
Smp_068950	histone binding protein Caf1
TCONS_00010310	hypothetical protein
Smp_005790	guanine nucleotide binding protein (G protein)
Smp_082280	RNA binding motif single stranded interacting
TCONS_00012108	EF-hand calcium-binding domain-containing protein 2
Smp_092180	RPA interacting protein
TCONS_00005416	hypothetical protein
TCONS_00000097	KIF1-binding protein homolog
Smp_078700	hypothetical protein
Smp_078780	dihydropyrimidinase protein 1
Smp_202000	hypothetical protein
TCONS_00019729	missing oocyte, meiosis regulator, homolog (Drosophila)
Smp_182880	hypothetical protein
Smp_021340	max dimerization protein 3
Smp_105860	ski interacting protein
TCONS_00016138	coiled-coil domain containing 147
Smp_194950	ELAV protein 2
TCONS_00006612	hypothetical protein
Smp_165470	epidermal growth factor receptor
Smp_145370	hypothetical protein
Smp_083730	hypothetical protein
Smp_201950	hypothetical protein
TCONS_00006144	hypothetical protein
Smp_147910	hypothetical protein
TCONS_00020287	UDP-N-acteylglucosamine pyrophosphorylase 1, like 1
SmtRNA_01086_Pseudo_TTG.1.1	tRNA
Smp_073470	Nuclear Receptor subfamily - retinoid-x-receptor (RXR)
Smp_129900	sodium and chloride dependent GABA transporter
TCONS_00011893	Uncharacterized protein
Smp_017900	ribosomal protein S6 kinase, polypeptide 2
SmtRNA_01293_Gln_TTG.1.1	tRNA
Smp_008770	Mitochondrial 28S ribosomal protein S32
SmtRNA_01061_Pseudo_TTG.1.1	tRNA
Smp_123165	Dynactin subunit 4
