# Supplementary figures and images for: The Epigenome of Schistosoma mansoni Provides Insight about How Cercariae Poise Transcription until Infection
Source: PLoS Negl Trop Dis. 2015 Aug 25;9(8):e0003853. doi: 10.1371/journal.pntd.0003853 (PMC4549315; doi:10.1371/journal.pntd.0003853)

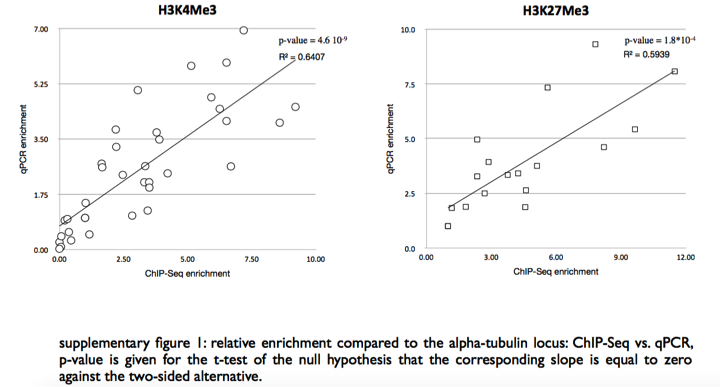

Supplement: S1 Fig — (TIFF) [file pntd.0003853.s005.tiff]
